# Supplementary material for: Implementation of a Delayed Prescribing Model to Reduce Antibiotic Prescribing for Suspected Upper Respiratory Tract Infections in a Hospital Outpatient Department, Ghana
Source: Antibiotics (Basel). 2020 Nov 4;9(11):773. doi: 10.3390/antibiotics9110773 (PMC7694150; doi:10.3390/antibiotics9110773)
Supplement: Supplementary file 1 [file antibiotics-09-00773-s001.pdf]

Supplementary Materials

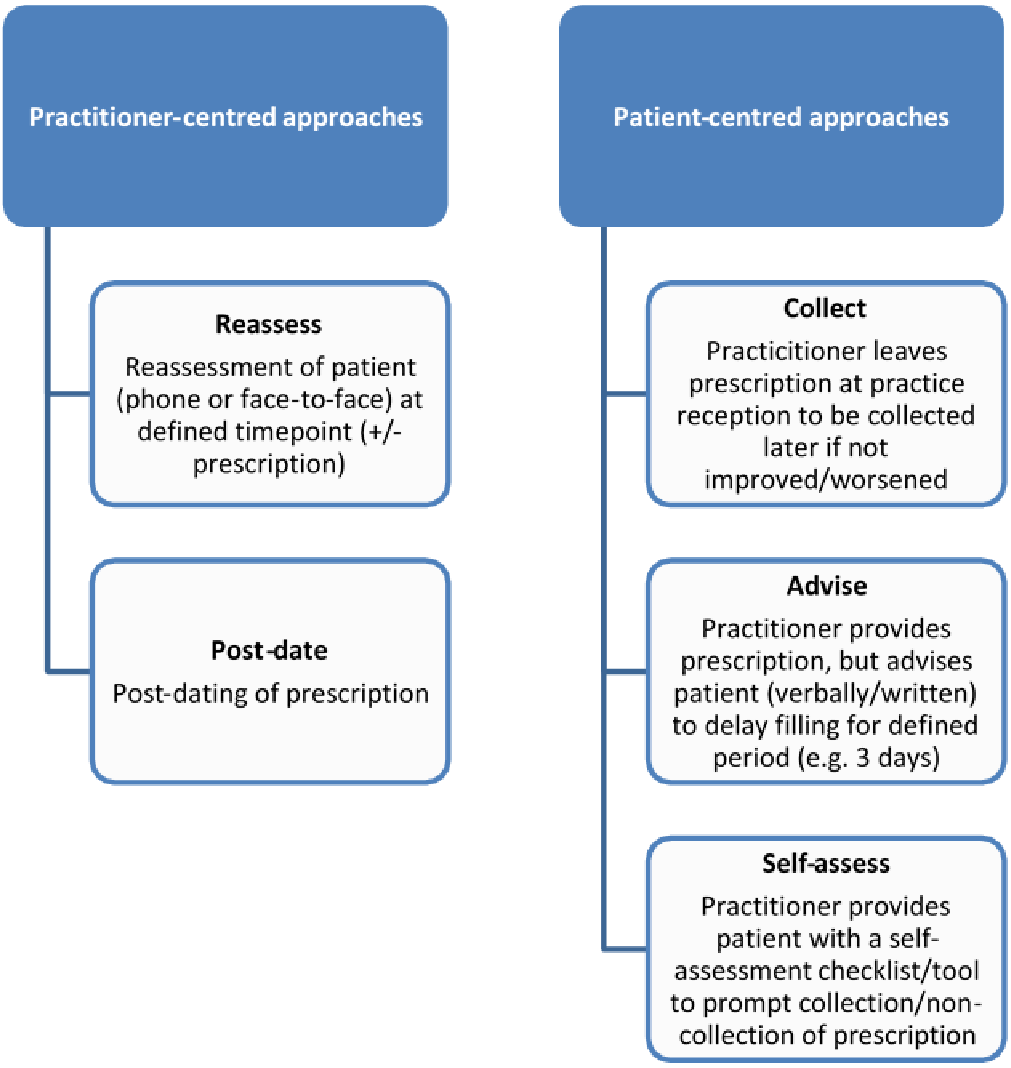

Figure S1. Typography of approaches to delayed antibiotic prescription.

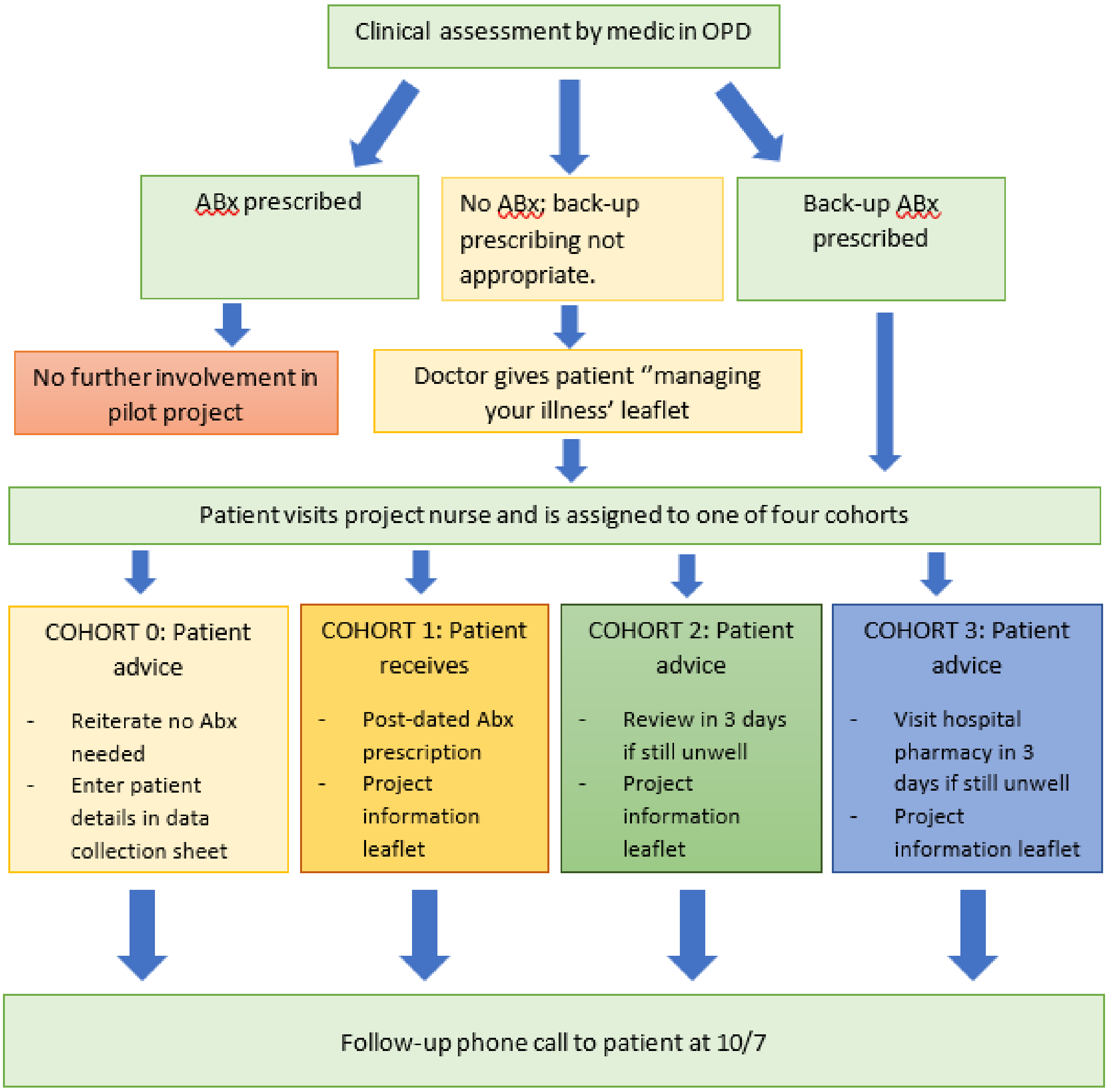

Figure S2. Patient pathways for project of delayed prescribing in LEKMA Hospital outpatients' department, Ghana.
